# A Tertiary Care Centre Experience of Recurrent Giant Cell Tumor Around the Knee Joint

**DOI:** 10.7759/cureus.29788

**Published:** 2022-09-30

**Authors:** Kshitish C Behera, Mohit Singla, Umesh Yadav, Majumdar KP, Tapish Shukla, Anand Gupta, Ajay Sheoran, Zile Singh Kundu, Ashish Devgun, Shagnik Paul

**Affiliations:** 1 Department of Orthopaedics, Homi Bhabha Cancer Hospital, Varanasi, IND; 2 Department of Orthopaedics, Pandit Bhagwat Dayal Sharma Post Graduate Institute of Medical Sciences, Rohtak, IND; 3 Department of Orthopaedics, Fortis Hospital, Mohali, IND; 4 Department of Surgical Oncology, Positron Multispecialty & Cancer Hospital, Rohtak, IND

**Keywords:** knee arthrodesis, gct recurrence, extended curettage, mega prosthesis, bone tumors

## Abstract

Introduction: Giant cell tumor (GCT) is a benign but locally aggressive bone tumor. It has a peak incidence between 30-40 years with a predilection for the epiphyseal/metaphyseal region of bone. The most common locations for bone GCT are the distal femur, proximal tibia, distal radius, and sacrum in decreasing order.

Material and Methods: In this retrospective study, 22 patients (13 females and nine males) with recurrent giant cell tumors around the knee joint between 2009-2022, with a mean age of 30.2 years (range: 18-55) were included. The patients were followed up monthly for three months, three-monthly for the next two years, six-monthly for the next five years, and thereafter, yearly. The mean follow-up period was 36.97 months (range 23-120 months).

Results: There were 19 recurrences after curettages and three after resections. Re-extended curettage was done in 17 cases and the resultant cavities were filled with autologous bone grafts in six and with polymethyl methacrylate (PMMA) cement in the other 11 cases. Reconstruction with megaprosthesis was done in two patients whereas knee arthrodesis was done in two patients after wide resection. The average Musculoskeletal Tumor Society (MSTS) score of our series of 22 patients was 23.1 (Range: 19-30).

Conclusion: Campanacci grade 1 and 2 lesions can be successfully treated with extended curettage and bone grafting/bone cementing. For patients with grade 3 lesions, there are two options available according to the financial status of the patient; the first option is reconstruction with prosthesis and the other option is arthrodesis.

## Introduction

Giant cell tumor (GCT) is a benign but locally aggressive bone tumor representing approximately 5% of all primary bone tumors [1]. It has a peak incidence between 30-40 years with a predilection for the epiphyseal/metaphyseal region of bone [2]. Metastasis occurs in 5% of cases and malignant transformation in 1-3% of cases [3-5]. The most common locations for bone GCT are the distal femur, proximal tibia, distal radius, and sacrum in decreasing order [6]. The GCT can extend up to subchondral bone and abuts the cartilage but the joint or its capsule is rarely invaded [7]. About 12% of patients present with a pathological fracture at the time of diagnosis [8]. Presence of pathological fracture points toward a more aggressive disease with a greater risk of metastasis spread and local recurrence [9]. As GCT is mostly located near joints and is usually benign in nature, many studies indicate an intralesional approach that preserves bony anatomy as compared to resection [10,11]. On other hand, many studies suggest that wide resection leads to decreased risk of local recurrence when compared with intralesional curettage [12,13]. But wide resection of tumors can lead to more surgical complications and functional impairment ultimately demanding reconstruction [14].

Surgical treatment of recurrent GCT around the knee joint is a controversial topic because of the difficulty in achieving a balance between reducing the chances of recurrence and preserving the knee joint function. Therefore, this retrospective study was undertaken to highlight the various surgical treatment options and functional and oncological outcomes related to them.

## Materials and methods

**Figure 1:**
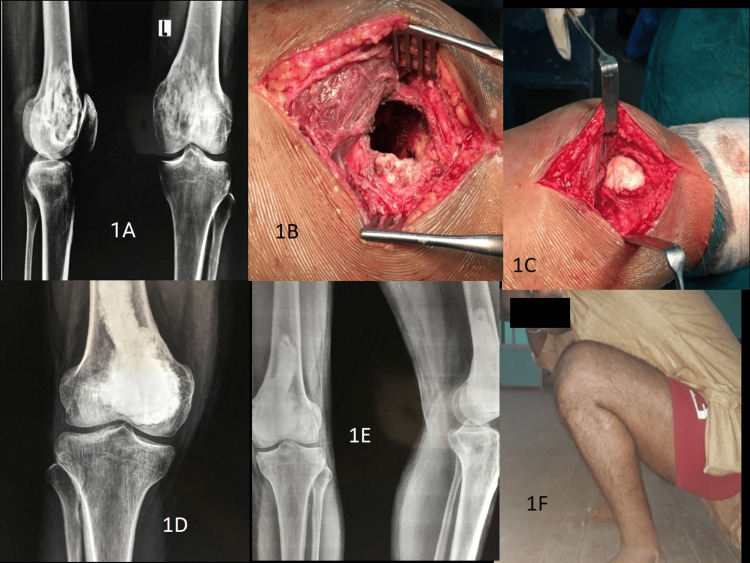
Recurrent GCT of distal femur in a 31-year-old male treated with extended curettage and bone cementing: (A) Preoperative radiograph showing recurrent GCT in distal femur; (B) Cavity after extended curettage; (C) Cavity filled with bone cement; (D) Postoperative radiograph showing extended curettage with bone cementing; (E) Follow-up radiograph after six months; (F) Functional outcome GCT: giant cell tumor

In this retrospective study, 22 patients (13 females and nine males) with recurrent GCTs around the knee joint between 2009-2022, with a mean age of 30.2 years (range: 18-55) were included. The orthopedic oncology clinic and the department of orthopedics provided access to the patients' records. The plain X-rays in two views (i.e. anteroposterior and lateral views) were studied in all the cases. The staging was carried out utilizing an MRI or CT scan of the local site along with a chest X-ray. The X-rays were assessed using Campanacci et al.'s radiological grading [15] and compartmental extension using Enneking et al.'s staging systems [16]. In conjunction with the operating surgeon, the radiographs were reviewed and studied by the musculoskeletal radiologist. The diagnosis was supported by a biopsy, which also excluded any malignant changes. All the recurrent lesions were benign GCTs and none showed malignant changes in this series. Recurrent GCTs of the distal femur in a 31-year-old male treated with extended curettage and bone cementing have been shown in Figure 1.

Follow-up

**Figure 2:**
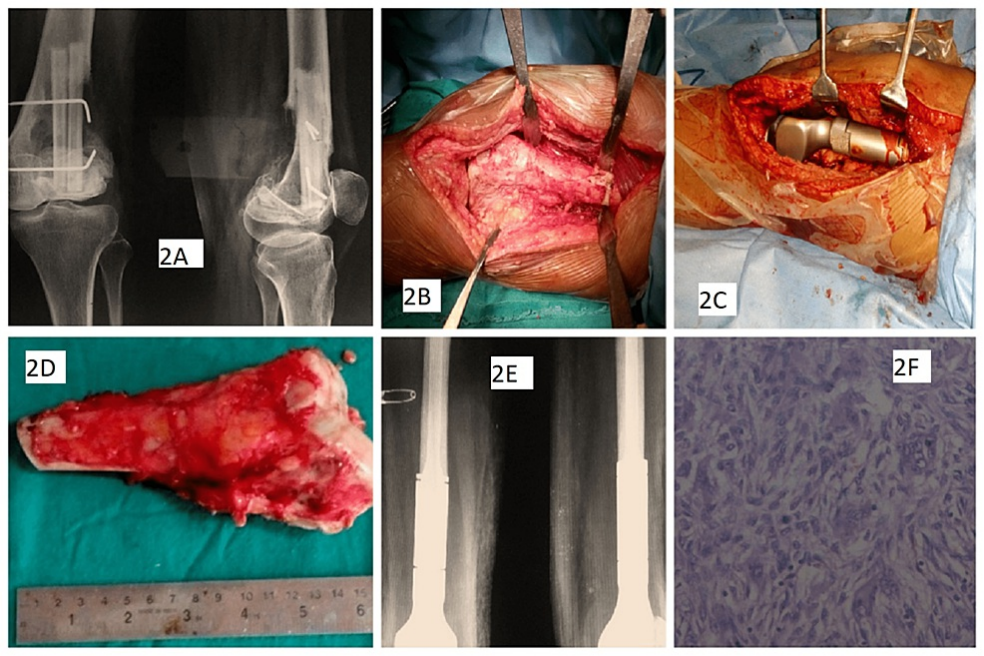
A female patient aged 24 years with distal femur GCT recurrence, which was treated with resection and megaprosthesis: (A) Preoperative radiograph showing extended curettage and bone grafting; (B) Intraoperative image showing involved area; (C) Intraoperative image showing megaprosthesis; (D) Resected specimen; (E) postoperative radiographs showing megaprosthesis; (F) Histological image of recurrent GCT (H&E stain) GCT: giant cell tumor;  H&E: hematoxylin & eosin

The mean follow-up period was 36.97 months (range 23-120 months). The plain X-rays of the local site and chest were done at three months, six months, one year, and thereafter, annually. A distal femur GCT recurrence that was treated with resection and megaprosthesis is shown in Figure 2.

## Results

**Figure 3:**
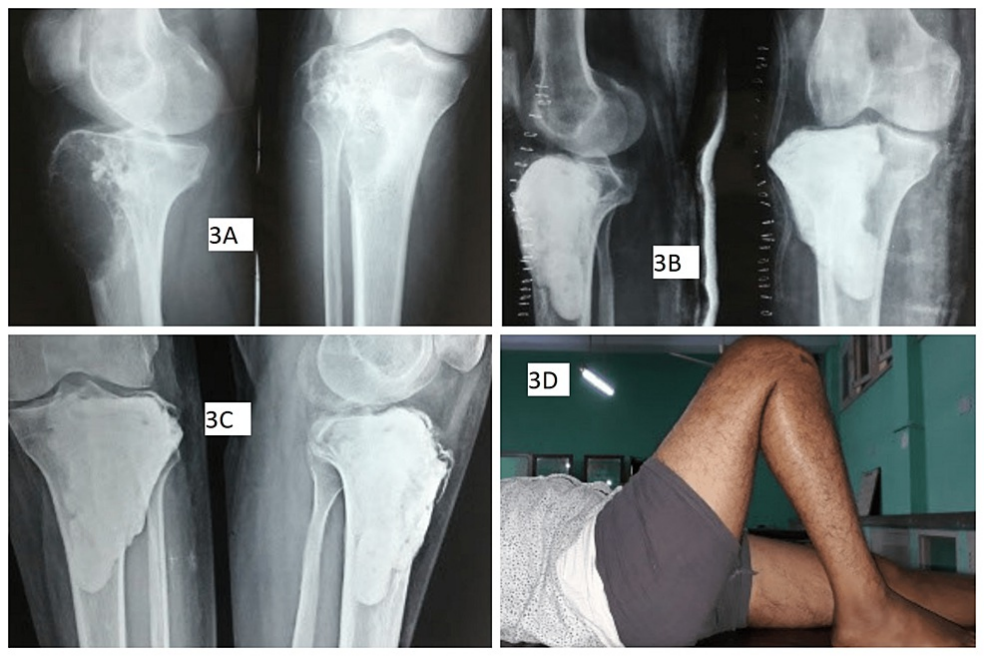
Recurrent GCT of proximal tibia in a 24-year-old male who was treated with extended curettage and bone cementing: (A) Preoperative radiograph showing recurrent GCT in proximal tibia; (B) Postoperative radiograph showing extended curettage with bone cementing; (C) Follow-up radiograph after six months; (D) Functional outcome GCT: giant cell tumor

Grading of tumors (Campanacci) and details of the patients are depicted in Table 1. There were 13 female and nine male patients. The mean age was 30.2 years (range: 18-55). Twelve recurrences were noticed within six months of the first surgery, six within one year, and four presented at 18 months after surgery. There were 19 recurrences after curettages and three after resections. Twenty patients with recurrences were treated initially outside by non-oncological orthopedic surgeons. These osseous recurrent lesions were graded as per Campanacci et al.'s radiologic grading as grade 1 (n=5, contained within the bone with no cortical expansion), grade 2 (n=12, lesions with expansion of the cortex), and grade 3 (n=5, lesions with extension into the soft tissues). Re-extended curettage was done in 17 cases and the resultant cavities were filled with autologous bone grafts in six and with polymethyl methacrylate (PMMA) cement in the other 11 cases. Recurrent GCT of the proximal tibia in a 24-year-old male who was treated with extended curettage and bone cementing has been shown in Figure 3.

**Table 1:** Patient demographic data

Variable	Number	Percent
Gender		
Male	9	40.9
Female	13	59.1
Grade (Campanacci) Femur		
Grade-I	2	9.09
Grade-II	7	31.8
Grade-III	3	13.6
Grade (Campanacci) Tibia		
Grade-I	3	13.6
Grade-II	5	22.7
Grade-III	2	9.09

**Figure 4:**
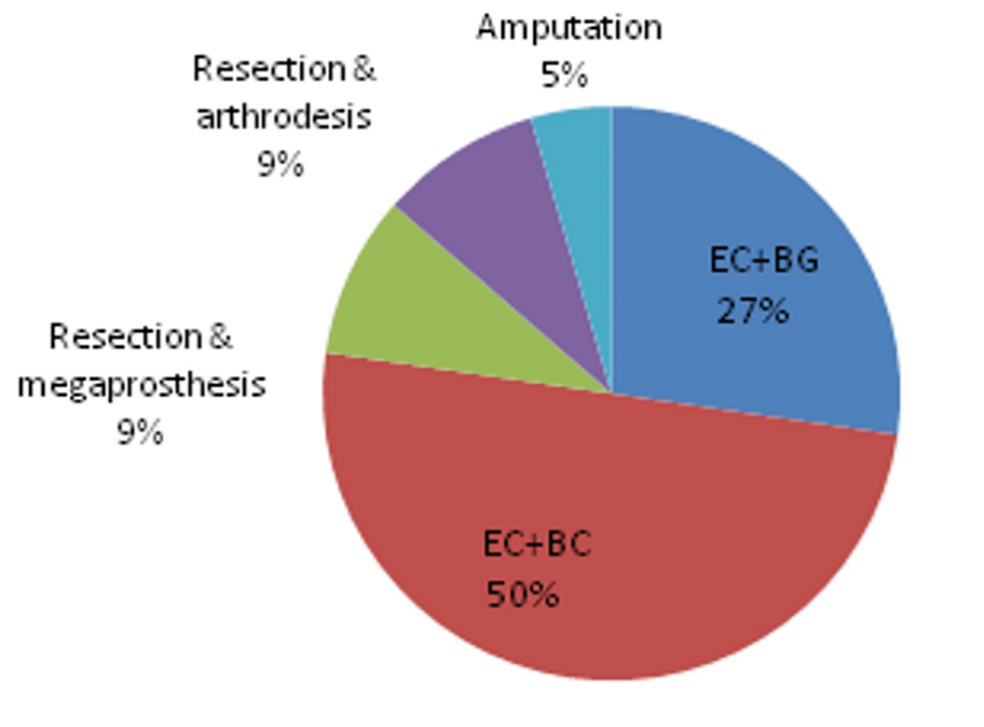
Various treatment modalities EC: extended curettage; BG: bone grafting; BC: bone cement

Four cases required resection of recurrence. Reconstruction with megaprosthesis was done in two patients while knee arthrodesis was done in two patients after wide resection. An intramedullary nail was used for the arthrodesis in these two patients. One patient with massive local recurrences with encasement of major neurovascular involvement was treated with above-knee amputation. Figure 4 illustrates the various treatment modalities used. 

Functional results

The functional status was assessed using the Musculoskeletal Tumor Society (MSTS) scoring system and Table 2 depicts the data regarding the same. The patients with joint preservations where further curettage was performed (n=13) had good functional outcomes with a mean MSTS score of 26.5 (Range: 22-30). The overall MSTS score of our series of 22 patients was 23.1 (Range: 19-30).

**Table 2:** Functional result according to MSTS score MSTS: Musculoskeletal Tumor Society

Type of Surgery	Number	Mean	Range
Joint preservation surgery	17	26.5	22-30
Wide resection and megaprosthesis	2	24.5	23-26
Wide resection and arthrodesis	2	22.5	22-23
Amputation	1	19	19
Overall	22	23.1	19-30

Complications

There was a superficial infection in one case, which was managed with intravenous antibiotics. Re-recurrence was reported in one case of recurrent distal femur GCT treated with intralesional curettage and bone grafting previously. Re-recurrence was managed with curettage and bone cementing. Joint stiffness in two patients was reported for which appropriate rehabilitation protocol was implemented.

## Discussion

GCTs of bone can arise at any location but distal femur and proximal tibia are the most common sites affected [17]. Earlier, the recurrence rate of GCT was 40-60% due to inappropriate surgical margins [18]. Extended curettage along with adjuvants has brought down the recurrence rate to less than 25%. Wide resection has brought it down to about 5% but with added morbidity [19]. When recurrence occurs after the curettage, X-rays can pick up the lysis that appears between the filler (bone graft/bone substitute or cement) and the host bone. This appearance of lysis can be picked up early when the cavity is being filled with bone cement due to its homogenous nature and larger rim of lysis as compared to cavities filled with bone grafts [20]. Capsular burst during resection can lead to spillage further leading to recurrence. In a study by Kivioja et al, 147 patients underwent intralesional curettage and bone cement filling, and recurrence was reported in 22% of cases, while the recurrence rate was 52% in 47 cases of curettage and bone grafting [21]. Vult von Steyern et al. treated 14 patients with repeat curettage and PMMA filling with good to excellent outcomes out of 19 cases of recurrent GCT [22]. The PMMA reduces the re-recurrence due to its thermal and toxic effects on tumor cells. In our study, intralesional curettage was done in Campanacci grade 1 and grade 2 patients (n=17). Only one re-recurrence was observed in this study, in which curettage and bone grafting was done.

Adequate exposure to the lesion is the key to ensuring complete removal of the tumor with an adequate curettage. A large cortical window is required to access the tumor so as to avoid curettage under overhanging shelves or ridges of bone. A high-power burr is useful in extending the curettage by breaking the bony bridges [23]. A pulsatile jet lavage system after the curettage helps in physically washing out tumor cells [24].

**Figure 5:**
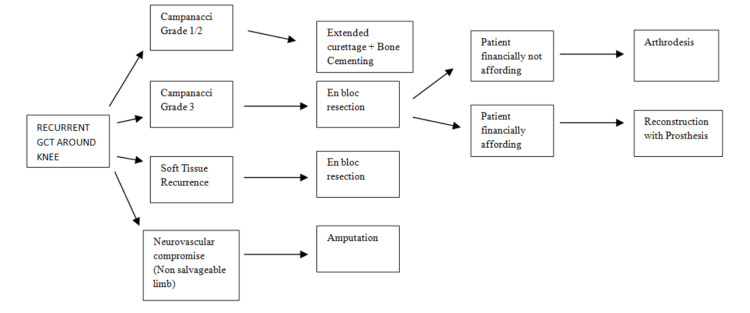
Treatment guidelines for patients with recurrent GCT around knee Image credits: Mohit Singla GCT: giant cell tumor

As the local behavior of GCT can be aggressive with a high tendency of recurrence, many authors prefer en bloc resection and reconstruction for grade 3 lesions in view of preventing local recurrence and joint function [25]. Natarajan et al. managed to achieve satisfactory oncological and functional outcomes by using the technique of limb salvage by custom megaprosthesis [26]. In our study, en bloc resection and reconstruction with megaprosthesis were done in two patients with a mean MSTS score of 24.5. Two patients were given the option of en bloc resection and reconstruction with megaprosthesis but due to financial constraints, en bloc resection and arthrodesis were done. In comparison to curettage and cementing/bone grafting, cases with wide resection and reconstruction have higher chances of facing difficulties like prosthesis loosening, infection, etc. Yu et al. studied 19 patients of en bloc resection and reconstruction with prosthesis, out of which prosthesis fracture and loosening developed in one, prosthesis aseptic loosening in three with prosthesis loosening rate of 31.6% [27]. As per our experience regarding the treatment and outcome of recurrent GCT around the knee, we propose the treatment guidelines presented in Figure 5.

Because the tissue planes are less distinct after recurrence, it is necessary to be careful when dissecting significant neurovascular structures in cases that have already undergone resection. But each lesion can be effectively treated with a specific method, such as extended curettage followed by the placement of cement or bone grafts, or excision and reconstruction according to the lesion’s grade and stage, much like with primary lesions. The functional results did not substantially worsen after the second surgery. According to the histopathology of these lesions after recurrence, none of the lesions changed in biological behavior and all remained benign.

Primary care of these cases by conventional orthopedic surgeons who are not specifically trained in orthopedic oncology has a major impact on the recurrence rate. In our study, 20 patients were referred by general orthopedic surgeons. Therefore, we advise that the recurrences be handled carefully, and preferably by an orthopedic surgeon who has had training in oncology. In experienced hands, the likelihood of recurrence does not rise if these are further sufficiently curetted or excised according to the degree of the lesion. However, in comparison to primary de novo lesions, further treatment in recurrent lesions with curettage or excision becomes challenging and should be handled by a person with expertise in this field due to the scarring and fudging of tissue planes. The advantage of this study is that all cases were handled by a single orthopedic oncological surgeon and the same team in a single institution. In a reasonably large series of 22 patients, there is a good follow-up period of 2-10 years. In conjunction with the operating surgeon, each case was examined histopathologically by pathologists with musculoskeletal onco-pathology training, and radiologically by an experienced radiologist. The fact that 20 patients were initially treated by general orthopedic surgeons elsewhere and referred to us after a recurrence is one of the study’s limitations. It is unclear if many of these cases actually involved microscopic illness or a residual macroscopic tumor. It is unknown how well the curettage has been executed. Since we have included cases of recurrence after curettage as well as after resection, there is no comparison between the cases in light of prior treatment.

## Conclusions

Campanacci grade 1 and 2 lesions can be successfully treated with extended curettage and bone grafting/bone cementing. For patients with grade 3 lesions, there are two options available according to the financial status of the patient, i.e. reconstruction with prosthesis and other option is arthrodesis. In addition to the various modalities of treatment, it is necessary that these lesions be primarily managed by orthopedic surgeons with adequate training in this field to lower the chances of recurrence.
